# Supplementary material for: Behind the smile: qualitative study of caregivers’ anguish and management responses while caring for someone living with heart failure
Source: BMJ Open. 2017 Jul 20;7(7):e014126. doi: 10.1136/bmjopen-2016-014126 (PMC5577885; doi:10.1136/bmjopen-2016-014126)
Supplement: Supplementary file 1 [file bmjopen-2016-014126supp001.docx]

**Research Checklist COREQ**

| 1. Interviewer/facilitator | **Which author/s conducted the interview or focus group?**  Jennifer Wingham and Dave Turner  Page 5 and 15 |  |
| --- | --- | --- |
| 2. | **Credentials**  *PhD for all authors*  Page 5 | What were the researcher's credentials? *E.g. PhD, MD* |
| 3. | ***Occupation***  *JW – Senior Clinical Researcher*  *JF Lecturer*  *NB Professor of Health Research*  *Title page* | What was their occupation at the time of the study? |
| 4. | **Gender**  All authors are female. One data collector was male.  Page 5 | Was the researcher male or female? |
| 5. | **Experience and training**  *All experienced qualitative researchers with published papers. NB and JF have published qualitative methodology papers*  *Page 5* | What experience or training did the researcher have? |
| Relationship with participants | None  Page 5 |  |
| 6. | **Relationship established**  *Contact was made with each participant by telephone to introduce the study once the participant had returned a screening questionnaire they had received from a clinician. Interviews were conducted face to face.*  *Reported in previous study referenced in methods page 4* | Was a relationship established prior to study commencement? |
| 7. | ***Participant knowledge of the interviewer***  *All participants were informed about the purpose to develop a manual for caregivers of people living with heart failure Page 4 –informed consent* | What did the participants know about the researcher? e*.g. personal goals, reasons for doing the research* |
| 8. | ***Interviewer characteristics***  *JW is a nurse, previous work was declared in the introduction.*  *Pages 4 and 5* | What characteristics were reported about the interviewer/facilitator? e.g. *Bias, assumptions, reasons and interests in the research topic* |
| **Domain 2: study design** |  |  |
| Theoretical framework |  |  |
| 9. | **Methodological orientation and Theory**  *This was informed by thematic analysis*  *Page 5* | What methodological orientation was stated to underpin the study? *e.g. grounded theory, discourse analysis, ethnography, phenomenology, content analysis* |
| Participant selection |  |  |
| 10. | **Sampling**  *Purposive sampling*  *Page 4* | How were participants selected? *e.g. purposive, convenience, consecutive, snowball* |
| 11. | **Method of approach**  Reported in previous paper reference 9  Page 4 | How were participants approached? e*.g. face-to-face, telephone, mail, email* |
| 12. | **Sample size**  *22*  *Abstract and 6* | How many participants were in the study? |
| 13. | **Non-participation**  *One was not contactable and one other changed her mind.*  *Not reported* | How many people refused to participate or dropped out? Reasons? |
| Setting |  |  |
| 14. | **Setting of data collection**  *Data was collected in the home of participants except for one who was interviewed in the research unit at their request.*  *Page 6* | Where was the data collected? e*.g. home, clinic, workplace* |
| 15. | **Presence of non-participants**  *In some of the interviews, the cared for person was present. This was at the request of the caregiver.*  *Page 6* | Was anyone else present besides the participants and researchers? |
| 16. | **Description of sample**  *3 areas in England. Date of interviews all in 2013. Demographic details were summarised.*  *Page 6* | What are the important characteristics of the sample? *e.g. demographic data, date* |
| Data collection |  |  |
| 17. | **Interview guide**  *Interview guide was developed with a PPI group. Topics are reported in the paper*  *Page 5* | Were questions, prompts, guides provided by the authors? Was it pilot tested? |
| 18. | **Repeat interviews**  *No interviews were repeated* | Were repeat interviews carried out? If yes, how many? |
| 19. | **Audio/visual recording**  *Interviews were audio-recorded*  *Pages 4 and 5* | Did the research use audio or visual recording to collect the data? |
| 20. | **Field notes**  *Field notes were written straight after the interviews*  *Page 5* | Were field notes made during and/or after the interview or focus group? |
| 21. | **Duration**  *Interviews lasted between 42 and 87 minutes*  *Page 6* | What was the duration of the interviews or focus group? |
| 22. | **Data saturation**  This study was an analysis of existing data and therefore there may be other themes not represented here.  Page 14 - Limitation | Was data saturation discussed? |
| 23. | **Transcripts returned**  *Transcripts were offered to participants. All were informed of the results by telephone and letter.*  *Page 6* | Were transcripts returned to participants for comment and/or correction? |
| **Domain 3: analysis and findings**z |  |  |
| Data analysis |  |  |
| 24. | **Number of data coders**  *Three data coders*  *Page 5* | How many data coders coded the data? |
| 25. | **Description of the coding tree**  *A model is presented in the paper.*  Page 19 | Did authors provide a description of the coding tree? |
| 26. | **Derivation of themes**  No. A strength of this study is that the findings were unexpected.  Page 4 | Were themes identified in advance or derived from the data? |
| 27. | **Software**  NVIVO 10  Page 5 | What software, if applicable, was used to manage the data? |
| 28. | **Participant checking**  *No participants provided feedback but they were sent results of the needs of caregivers. The PPI group is aware of the findings of this study.*  *Page6* | Did participants provide feedback on the findings? |
| Reporting |  |  |
| 29. | **Quotations presented**  Yes | Were participant quotations presented to illustrate the themes / findings? Was each quotation identified? e*.g. participant number* |
| 30. | **Data and findings consistent**  *Yes* | Was there consistency between the data presented and the findings? |
| 31. | **Clarity of major themes**  *Yes* | Were major themes clearly presented in the findings? |
| 32. | **Clarity of minor themes**  *Yes Includes examples where participants were coping to illustrate what was different.* | Is there a description of diverse cases or discussion of minor themes? |
